# Large language models and generative AI in telehealth: a responsible use lens

**DOI:** 10.1093/jamia/ocae035

**Published:** 2024-03-04

**Authors:** Javad Pool, Marta Indulska, Shazia Sadiq

**Affiliations:** ARC Industrial Transformation Training Centre for Information Resilience (CIRES), The University of Queensland, Brisbane 4072, Australia; School of Electrical Engineering and Computer Science, The University of Queensland, Brisbane 4072, Australia; ARC Industrial Transformation Training Centre for Information Resilience (CIRES), The University of Queensland, Brisbane 4072, Australia; Business School, The University of Queensland, Brisbane 4072, Australia; ARC Industrial Transformation Training Centre for Information Resilience (CIRES), The University of Queensland, Brisbane 4072, Australia; School of Electrical Engineering and Computer Science, The University of Queensland, Brisbane 4072, Australia

**Keywords:** telehealth, artificial intelligence, large language models, ChatGPT, responsible use

## Abstract

**Objective:**

This scoping review aims to assess the current research landscape of the application and use of large language models (LLMs) and generative Artificial Intelligence (AI), through tools such as ChatGPT in telehealth. Additionally, the review seeks to identify key areas for future research, with a particular focus on AI ethics considerations for responsible use and ensuring trustworthy AI.

**Materials and Methods:**

Following the scoping review methodological framework, a search strategy was conducted across 6 databases. To structure our review, we employed AI ethics guidelines and principles, constructing a concept matrix for investigating the responsible use of AI in telehealth. Using the concept matrix in our review enabled the identification of gaps in the literature and informed future research directions.

**Results:**

Twenty studies were included in the review. Among the included studies, 5 were empirical, and 15 were reviews and perspectives focusing on different telehealth applications and healthcare contexts. Benefit and reliability concepts were frequently discussed in these studies. Privacy, security, and accountability were peripheral themes, with transparency, explainability, human agency, and contestability lacking conceptual or empirical exploration.

**Conclusion:**

The findings emphasized the potential of LLMs, especially ChatGPT, in telehealth. They provide insights into understanding the use of LLMs, enhancing telehealth services, and taking ethical considerations into account. By proposing three future research directions with a focus on responsible use, this review further contributes to the advancement of this emerging phenomenon of healthcare AI.

## Introduction

The integration of Artificial Intelligence (AI)-enabled services in healthcare systems has the potential to transform digital health business models. Large Language Models (LLMs) and generative AI tools, such as ChatGPT, have received significant attention due to their potential to transform healthcare services and augment clinical decision support.^1–6^ These advanced AI models possess the ability to generate human-like text in response to prompts, engage with users in natural language conversations, and offer decision support for informed action.

In the context of telehealth, the effective use of LLMs holds promise and potential for service innovation. Telehealth is the practice of delivering healthcare services remotely through electronic information and telecommunication systems. It includes real-time audio and visual connections between patients and health professionals, remote patient monitoring, and virtual check-ins via various communication tools.^7^ In traditional telehealth practices interactions between patients and clinicians often require both parties to be present simultaneously, limiting flexibility. However, with the potential synergy between telehealth and LLMs, a compelling dimension emerges. LLMs have the potential to assist healthcare providers as clinician-AI assistants and chatbots during telehealth, which in turn could lead to better augmentation of e-consultations in synchronous and also asynchronous scenarios. This capability holds promise for more efficient patient care delivery, streamlined workflows, facilitated information exchange, and enhanced patient experience. For instance, Cheng et al.^8^ noted that by incorporating ChatGPT into telehealth platforms, patients can benefit from improved quality of care and remote guidance provided by healthcare providers. These AI-enabled telehealth services also offer a unique opportunity to bridge communication challenges concerning health literacy, empathy, and language barriers, empowering both healthcare providers and patients.^9^^,^^10^ By realizing the potential of LLMs-enabled telehealth services, health service providers have the opportunity to improve service quality and accessibility. However, a lack of appropriate AI regulations, incorrect outputs, privacy, and ethical concerns create challenges for its responsible use.^11–17^

While the use of LLMs in telehealth shows promise, there is a need to conduct a scoping review to assess the current state of research. Such a review allows identification of knowledge gaps so that research can more systematically advance. Accordingly, our paper presents the first scoping review that specifically focuses on LLM applications in telehealth, offering a systematic analysis of existing literature. Further, we seek to propose future research directions with a particular focus on the ethical lens for trustworthy AI. To ensure the responsible use and integration of these technologies in telehealth, we adopt the lens of AI ethics.^18^^,^^19^ In the telehealth context, the conceptual underpinnings of AI ethics center on ensuring that AI systems adhere to fundamental principles of trustworthiness, such as transparency and explainability. These principles emphasize legality, human value, and robustness. The responsible use of AI in healthcare, guided by AI ethics, supports beneficial telehealth services by ensuring safe and trustworthy AI, enhancing service delivery, and fostering trust between patients and telehealth providers. For instance, transparency builds trust and encourages patients to engage more with telehealth services. For healthcare providers, clear explanations from generative AI systems can help them understand and trust the health recommendations they receive, potentially leading to better clinical decisions and consequently improving patient outcomes. These trustworthy AI systems help make telehealth a more reliable and beneficial option for healthcare stakeholders. With this responsible use lens, we explore the opportunities and challenges related to ethical considerations, transparency, data privacy, and the potential consequences that may arise from LLMs use in telehealth.

## Methods

To conduct this scoping review, we followed a methodological framework established by Arksey and O'Malley.^20^ This framework consists of 6 stages: (1) “identifying the research question”, (2) “identifying relevant studies”, (3) “study selection”, (4) “charting the data”, (5) “collating, summarizing, and reporting the results”, and optionally (6) “expert consultation”. In our review, we conducted the first 5 steps, and omitted the optional step due to the emerging nature of the topic. A similar approach has been adopted in recent investigations in medical informatics research,^21^^,^^22^ particularly in the domain of healthcare AI.^23–25^ Given the emerging status of LLMs applications in telehealth and the relative novelty of implementing such technology on a wide scale, accessing relevant experts is challenging. Our focus on providing a comprehensive overview across various concepts within the responsible use of LLMs in telehealth prioritized breadth over specific expert input.

In the first stage, the research question was formulated to explore “What is the current state of research on the responsible use of LLMs in telehealth, and what are the knowledge gaps in this context of use?” The specific question was to gain insights into the current research landscape and highlight areas requiring further investigation, particularly in relation to responsible AI.

To identify relevant studies, we developed a search strategy using a combination of keywords and search terms related to telehealth, generative AI, ChatGPT, and LLMs. Inclusion criteria comprised English-language publications of empirical and non-empirical nature, encompassing reviews, perspectives, viewpoints, and conference articles within the context of telehealth and systems involving LLMs. Exclusion criteria consisted of non-English papers, and abstract-only papers. Table 1 summarizes the research method, including the research question, search strategy, databases searched, and inclusion and exclusion criteria.

**Table 1.** Overview of the research method.

Characteristic	Description
Research question	What is the current state of research on the responsible use of LLMs in telehealth, and what are the knowledge gaps in this context of use?
Search strategy	A combination of keywords and search terms related to telehealth, generative AI, ChatGPT, and LLMs were used to identify relevant studies. For telehealth, the keywords used included telehealth OR telemedicine OR telecare OR teleconsult* OR teledermatology OR telemental OR telerehabilitation OR telehomecare OR telegeriatric* OR telecardiology OR teleradiology. For generative AI and LLMs, the search terms used were ChatGPT OR “Generative pre-trained transformer” OR “generative artificial intelligence” OR “generative AI” OR “GPT-3” OR “GPT-3.5” OR “GPT-4” OR “GPT model*” OR “large language model*” OR LLMs OR Bard OR OpenAI OR “Google AI”.
Records identification	Scopus, Web of Science, CINAHL, Embase, IEEE Explore, PubMed, arXiv and Google Scholar.
Inclusion criteria	English-language publications, empirical and non-empirical research, encompassing reviews, perspectives, viewpoints, and conference articles within the context of telehealth and systems involving large language models, ChatGPT, and generative AI. Our inclusion strategy was not limited solely to peer-reviewed articles. We also included academic papers in the screening and eligibility stages of the review from repositories of electronic preprints and post-prints such as arXiv.org.
Exclusion criteria	Non-English papers, and abstract-only papers, grey literature (e.g, newsletters, technical reports/standards, blogs, government reports).
Data charting and reporting the results	Data charting involves organizing scoping review findings into a thematic structure. We employed the EU and Australia's AI ethics principles as a concept matrix. This framework aids in reporting and analyzing responsible use of LLMs concepts in health informatics literature.

## Results

**Figure 1.**
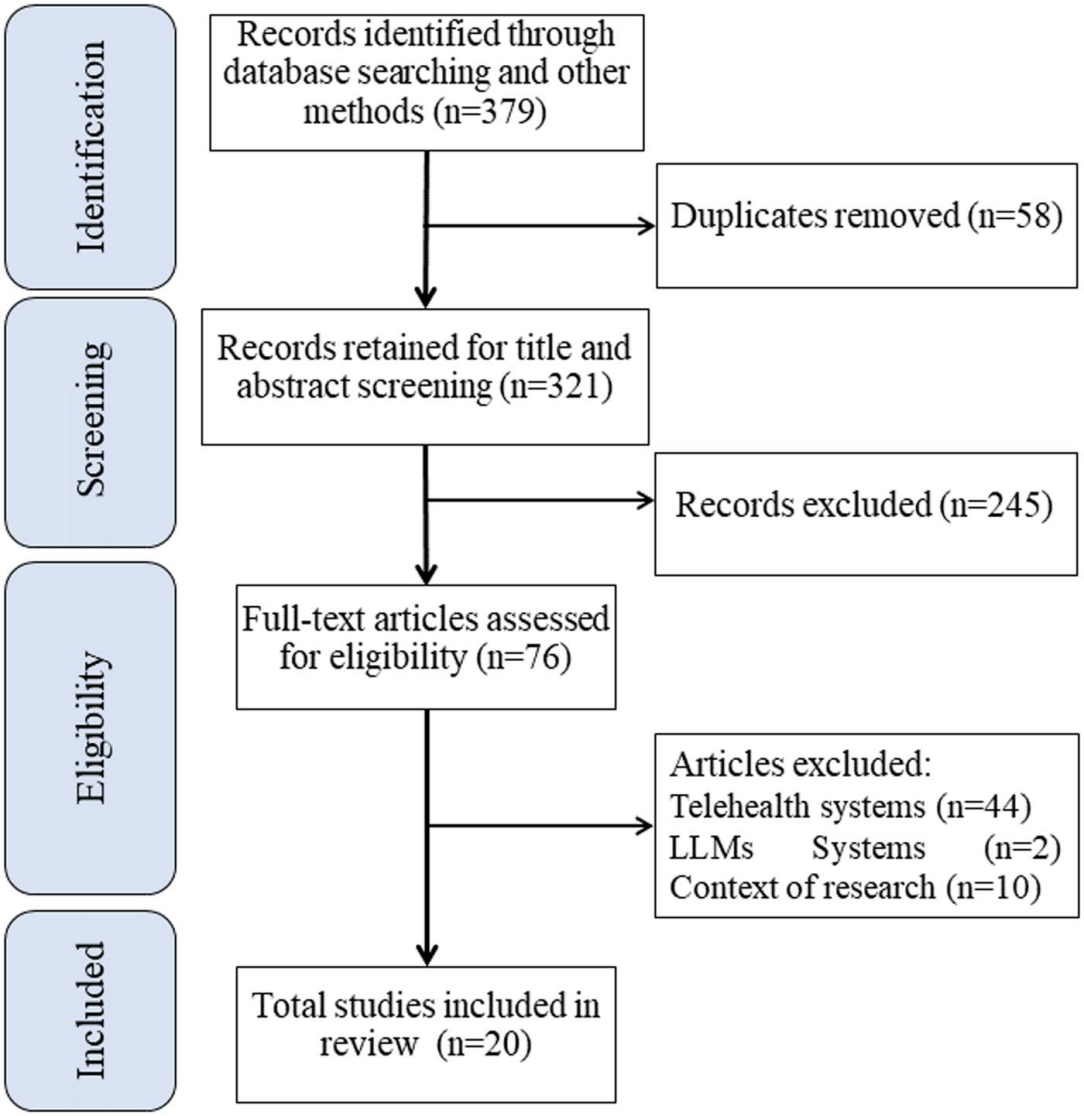
Study selection.

The search yielded a total of 379 identified records, from which 58 duplicates were removed, resulting in 321 unique records for screening. The screening process involved assessing the titles and abstracts of these records, leading to the selection of 76 articles for full-text review. In the full-text review stage, 20 studies met the inclusion criteria and were incorporated into the scoping review. The limited number of relevant studies meeting inclusion criteria reflects the nascent stage of research LLMs, generative AI and ChatGPT. Figure 1 illustrates the study selection process.

The included studies consist of 20 articles published in 2023. Notably, ChatGPT's first demo release in late 2022 may have influenced publication timelines, with no relevant publications identified before 2023. The data charting, summarized in the Table 2, highlights key attributes of the included articles. Five papers were empirical—three qualitative and two quantitative—focusing on the application of telehealth chatbots, teleconsultations, teleradiology, and the use of telehealth in aged care. Among the five articles, two were in the US context,^26^^,^^27^ two in South Korea,^28^^,^^29^ and one in Saudi Arabia.^30^ The other 15 articles are non-empirical, including 13 studies that provide a letter-style commentary, opinions or perspective on telehealth in general, and in specific contexts such as rural healthcare, mental health, and surgery. The remaining three papers were review papers focused on telehealth within the context of dental medicine, psychiatry and oncology.

**Table 2.** Overview of included articles.

Authors	Type of Article	Empirical/non-empirical	Method	Telehealth context
Jeong et al.^28^	Conference	Empirical	Quantitative	Application of chatbot for telehealth
Alanzi^30^	Journal	Empirical	Qualitative	Teleconsultations
Huang et al.^26^	Journal	Empirical	Quantitative	Teleradiology in emergency care
Jo et al.^29^	Conference	Empirical	Qualitative	Application of chatbot for telehealth
Yang, et al.^27^	Preprint	Empirical	Qualitative	Telehealth in aged care
Snoswell et al.^31^	Journal	Non-empirical	Letter/viewpoint	Telehealth in general
Lahat and Klang^32^	Journal	Non-empirical	Letter/viewpoint	Telehealth in general
Eggmann et al.^33^	Journal	Non-empirical	Review	Telehealth in dental medicine
Meskó^34^	Journal	Non-empirical	Letter/viewpoint	Telehealth in general
Lawson McLean^35^	Journal	Non-empirical	Letter/viewpoint	Telehealth in general
Ahmed et al.^36^	Journal	Non-empirical	Letter/viewpoint	Telehealth in rural healthcare
Cheng et al.^37^	Journal	Non-empirical	Review	Telepsychiatry/telemental health
Liu et al.^38^	Journal	Non-empirical	Letter/viewpoint	Telehealth in general
Sarma et al.^39^	Journal	Non-empirical	Review	Telehealth in oncology
Vahedifard et al.^40^	Preprint	Non-empirical	Letter/viewpoint	Telepsychiatry/telemental health
Wang et al.^41^	Journal	Non-empirical	Letter/viewpoint	Telehealth in general
Prazeres^42^	Journal	Non-empirical	Letter/viewpoint	Telehealth in rural healthcare
Cox et al.^43^	Journal	Non-empirical	Letter/viewpoint	Telehealth in surgery
Srivastav et al.^44^	Journal	Non-empirical	Letter/viewpoint	Telehealth in radiology and medical imaging diagnosis
Alofi^45^	Journal	Non-empirical	Letter/viewpoint	Telehealth in general

Scoping review studies require an analytical framework or thematic structure to convey a narrative summary of the current body of literature.^20^ Given our aim of literature review in terms of responsible use of LLMs in telehealth, we draw upon the European Union (EU) ethics guidelines for trustworthy AI^46^ and Australia’s AI ethics principles^47^ to construct a concept matrix of responsible use (see Table 3). We select these frameworks because of their comprehensive coverage of responsible AI.^48^^,^^49^ Both the EU guidelines and Australia’s principles share similarities in their coverage of human-centered values and the mitigation of AI risks. The EU guidelines outline key requirements for trustworthy AI systems, emphasizing human agency, technical robustness, transparency, and societal well-being.^46^ Similarly, Australia’s principles also highlight human-centered values, fairness, privacy protection, contestability, and accountability, ensuring positive AI outcomes for individuals, society, and the environment.^47^ Building upon these ethical principles of trustworthy AI,^46^^,^^47^ together with the concept of system use,^50^^,^^51^ we define responsible use of AI as using an AI system in a way that is ethical and trustworthy, contributing to the achievement of the intended goals for using the AI system. Our definition encompasses users engaging with AI systems to perform tasks, and covers use-related activity and associated organizational practices that facilitate responsible use. The concept matrix serves as a tool for assessing the exploration of responsible use of AI concepts within the health informatics literature. Our approach aligns with recommendations for writing a concept-centric literature review.^52^ We specifically investigate how these concepts are investigated in the context of LLMs and generative AI, exemplified by systems like ChatGPT, within telehealth services.

**Table 3.** A concept matrix of responsible use of AI (adapted from European Commission^46^ and Australian Government^47^).

No.	Studies	Concepts									
		Beneficial outcomes	Fairness and inclusiveness	Privacy protection	Transparency and explainability	Contestability	Human agency	Reliability and safety	Security	Human oversight	Accountability
1	Jeong et al.^28^							✓			
2	Alanzi^30^	✓^a^		Peripheral^b^				Peripheral	Peripheral		Peripheral
3	Huang et al.^26^	✓						✓			
4	Jo et al.^29^	✓						✓			
5	Yang et al.^27^	✓		Peripheral				✓			
6	Snoswell et al.^31^	Peripheral	Peripheral					Peripheral			Peripheral
7	Lahat and Klang^32^	Peripheral	Peripheral					Peripheral			
8	Eggmann et al.^33^	Peripheral									
9	Meskó^34^	Peripheral									
10	Lawson McLean^35^	Peripheral	Peripheral					Peripheral	Peripheral		
11	Ahmed et al.^36^	Peripheral									
12	Cheng et al.^37^	Peripheral						Peripheral			
13	Liu et al.^38^	Peripheral									
14	Sarma et al.^39^	Peripheral									
15	Vahedifard et al.^40^	Peripheral									
16	Wang et al.^41^	Peripheral									
17	Prazeres^42^	Peripheral									
18	Cox et al.^43^	Peripheral									
19	Srivastav et al.^44^	Peripheral									
20	Alofi^45^	Peripheral									

a The “✓” check mark in the table indicates that the respective concept of responsible use is addressed in detail, either empirically or conceptually.

b “*Peripheral*” in this review signifies the responsible use on the periphery, not central to the study. Although the paper references a concept related to responsible use, it lacks empirical measures to substantiate or evaluate responsible use adequately or properly conceptualize responsible use. The focus persists on a related but secondary aspect.

Beneficial outcomes is the most often explored concept in the included studies, as illustrated in Table 3. Fifteen papers discussed beneficial outcomes, although the concept was peripheral in the papers. The term “peripheral” in this review means discussions that were not the primary focus of the paper. For example, while Snoswell, et al.^31^ offer valuable insights on the benefit of LLMs in telehealth—ie, augmenting telehealth services, offering potential benefits such as generating health information and assisting in lifestyle interventions—they do not empirically examine the concept of benefit or provide a conceptualization. Eggmann, et al.^33^ indicated that ChatGPT and LLMs can enhance dental telemedicine by efficient patient data collection, symptom analysis, and diagnosis suggestions, improving accessibility, scalability, and language translation for effective consultations. Four studies investigate the concept of benefit in some depth.^26^^,^^27^^,^^29^^,^^30^ For instance, Jo et al.^30^ investigate the concept of benefit by examining its impact on users, teleoperators, and developers. The research reveals benefits in holistic understanding, workload offloading, loneliness mitigation, and emotional burden reduction. Users appreciate diverse conversation topics, while teleoperators find the system valuable for monitoring and supporting users efficiently. Huang et al.^26^ indicated benefits for clinical applications—a promising application of generative AI to augment physician decision-making in clinical settings, for chest radiograph reports. While these studies provide insights into the benefits of ChatGPT in telehealth settings, they only indirectly touch upon the concept of effective use and the potential for users to generate better-informed medical actions. They don’t explicitly investigate the mechanisms for the effective use of LLMs to create benefits for various telehealth stakeholders.

Reliability and safety emerged as the second most explored concept in the review. Nine papers discussed reliability and safety with four collecting empirical evidence.^26–29^ For instance, Jeong et al.^28^ proposed a question-and-answer (Q&A) application that integrates AI into healthcare systems and is capable of functioning in the telemedicine context. Specifically, the research involved implementing a BERT-GPT-2 algorithm for healthcare Q&A, with BERT as encoder and GPT-2 as decoder. While perplexity and loss were worse compared to GPT-2 alone, qualitative assessment indicated better understanding of conversation intent. This result sheds light on the importance of AI systems functioning reliably in healthcare contexts and highlights that evaluation of LLMs in telehealth requires careful consideration, extending beyond technical metrics like perplexity alone. Jo et al.^29^ indicated concerns about the reliability of an AI-driven CareCall system in detecting emergencies. Users desire a direct link to emergency services, but developers are hesitant due to uncertainty about the systems’ reliability in critical care situations, fearing potential failures.

Fairness and inclusiveness are peripherally discussed in three papers.^31^^,^^32^^,^^35^ Lahat and Klang^32^ and Snoswell et al.^31^ briefly indicated the importance of addressing LLMs' limitations and biases, while ensuring their ethical use in the integration within telehealth services. Lawson McLean^35^ highlighted the presence of algorithmic bias in telehealth settings, which may result in disparities in treatment, especially among marginalized populations.

Privacy protection is mentioned in two papers,^27^^,^^30^ with only one paper empirically examining privacy concerns.^27^ Yang et al.^27^ identified privacy concerns among older adults using voice assistants, a system capable of integrating LLM-powered technology. Participants expressed worry about accidentally disclosing sensitive information, while others felt reassured if the system adhered to regulations.

Security and accountability is discussed in four studies, all in a peripheral context. Alanzi^30^ referred complexity of safeguarding patients' data security when employing AI applications like ChatGPT, emphasizing the potential breach of privacy and data security as users input sensitive details to seek suggestions. Snoswell et al.^31^ emphasized the need for clinician awareness as consumers engage with LLMs in telehealth, cautioning that LLMs may surpass professionals in delivering e-consults without regulatory oversight or accountability, highlighting the importance of verifying the sources of health information during discussions with consumers. In Alanzi^30^ study, participants expressed a sense of accountability and liability for decisions made using ChatGPT in teleconsultations, but uncertainty arose regarding responsibility in case of errors, as the AI system's introduces ethical and legal complexities.

**Figure 2.**
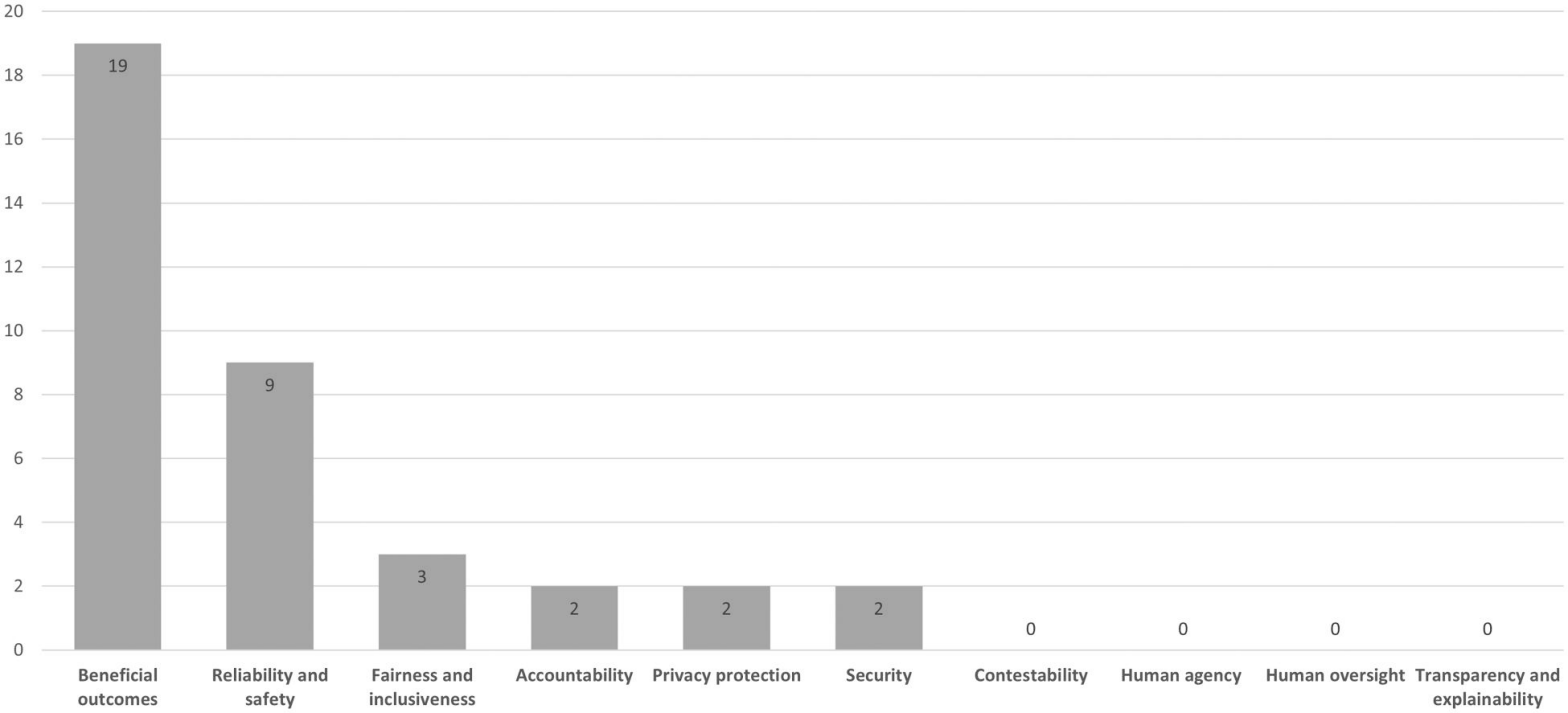
Distribution of responsible use concepts across included studies.

Notably, transparency and explainability, and human agency and oversight as well as contestability, are not examined empirically or conceptually in any of the included articles. The distribution of research studies across responsible use concepts is illustrated in Figure 2 (some studies explored multiple concepts).

## Towards responsible use of LLMs in telehealth

On the basis of our concept matrix, we identify three research directions to advance responsible use of LLMs in telehealth *viz. beneficial outcomes, human-centered values, and sociotechnical structural assurance.* While the first has a direct mapping to our beneficial outcomes concept, the latter two are research directions based on composites of concepts. Human-centered values is based on our analysis of five concepts, while sociotechnical structural assurance stems from our analysis of four concepts.

### Beneficial outcomes: enhancing health and wellbeing for all stakeholders

Responsible use of AI is crucial for maximizing its benefits while minimizing potential harm.^53^ By adopting a responsible approach, healthcare providers can use LLMs to improve telehealth services that are beneficial for ‘*enhanced health and wellbeing’* (the italicized, single-quoted phrases throughout the paper represent the concept of responsible use, drawing upon EU ethics guidelines and Australia’s AI ethics principles. While some phrases or words are verbatim quotations (e.g. ‘*transparency and explainability*’, and ‘*accountability’*), others are adaptations or summaries of the principles (e.g. ‘*enhanced health and wellbeing’ and ‘improved environmental sustainability’*) outlined in these ethical frameworks). Future research could explore the challenges and opportunities presented by LLM-enabled telehealth services for value creation and examine how they can effectively use AI to benefit patients and public health. Furthermore, future investigations could adopt effective use theory perspectives^51^ to identify and evaluate beneficial outcomes stemming from integrations LLMs in telehealth. Effective use theory provides an established theoretical foundation on which to evaluate how users leverage the system to achieve intended goals, particularly in terms of informed clinical actions—an essential aspect for ensuring responsible use of AI in healthcare contexts. Building on effective use theory,^51^^,^^54^ prospective studies could investigate the nuanced mechanisms of informed action by health professionals, identifying the specific practices and capabilities that contribute to achieving beneficial outcomes in individual performance, healthcare delivery and public health through the effective use of LLMs. In alignment, researchers could investigate several key aspects including personalized care, benefit for patients (receiver) and medical specialists (providers). For instance, future research can investigate how the integration of LLMs in healthcare systems can enhance the delivery of personalized care via telehealth. Developing and implementing LLM-enabled telehealth that meets individual patient needs while considering factors like cultures, language barriers, and socio-economic disparities is another valuable avenue for telehealth researchers who focus on design science. Future investigations should also explore the impact of LLMs and generative AI on the skills and decision-making processes of telehealth providers. Understanding how LLMs and ChatGPT influence the skills and practices of medical specialists is vital for ensuring not only positive health outcomes for patients but also the professional development and job satisfaction of healthcare providers. This inclusive approach aligns with the overarching trustworthy AI principles,^46^ benefiting all stakeholders in the telehealth ecosystem. While literature acknowledges the potential of LLMs in telehealth for patients and providers, in particular, applications like ChatGPT, gaps exist in empirically investigating their actualized benefits across stakeholders. Future research should detail the direct impact of LLMs on clinical practice, including their effects on workflows, decision-making processes, patient outcomes, and the well-being of clinicians. Detailed analysis can inform the effective use of telehealth, ensuring that responsible use of LLM leads to tangible improvements in healthcare delivery, provides better quality of health for patient care, and enhances the overall experience for clinicians.

It is also important to consider the environmental impact of LLMs-enabled telehealth services, including ‘*improved environmental sustainability’*. Telehealth use can play a crucial role in addressing the pressing challenge of climate change.^55–57^ A scoping review focusing on the role of digital health in reducing carbon emissions highlighted that telehealth contributes to lowering carbon footprint by minimizing patient and clinical staff travel.^58^ Telemedicine, combined with AI and Internet of Things (IoT), could potentially offer sustainable solutions to decrease the environmental impact of healthcare.^55^^,^^59–61^ In addition to the contributions of telehealth in mitigating climate change, the increased use of telehealth, as a result of the acceptance and effective use of LLMs, has the potential to further enhance sustainability in telehealth processes. By automating appointment scheduling, augmenting medical tasks during telehealth use, and minimizing the reliance on paper records, the use of LLMs can support to reduce energy consumption and further reinforce the positive impact of telemedicine on climate change mitigation. However, due to the limited research available, further investigation is necessary to evaluate the impact of this emerging technology on environmental wellbeing. Future telehealth research can also contribute to the United Nations’ environmental program on digital transformation by focusing on key areas such as climate, nature, and pollution.^62^ In line with this program, understanding how employing advanced AI analytics and LLMs in telehealth practices can expedite environmental sustainability could be a fruitful avenue for future investigation.

### Human-centered values and rights

Irresponsible use and improper implementation of LLMs create misalignment with clinical and societal values and rights. For instance, like other AI models, there is a risk that a LLM may incorporate biases (eg, racial or ethnic bias) that could exacerbate health inequities.^63–67^ LLMs in telehealth should be designed and used with a focus on human-centered values and rights, ensuring responsible use. These values and rights, which prioritize autonomy and fundamental rights of AI stakeholders such as domain experts (health professionals) and telehealth end users (patients), are grounded in ethical principles. We highlight five key aspects that encompass human-centered approaches: human agency, transparency and explainability, fairness and inclusiveness, privacy protection, and contestability.

Regarding ‘*human agency*’, it is crucial to investigate how improper automation, use, and over-reliance on LLMs in telehealth can impact the autonomy of both patients and telehealth providers. This investigation could inform designing responsible AI-enabled telehealth services, and minimizing the potential limitations of LLMs on decision-making freedom that may arise. Future research in the context of telehealth can also focus on exploring mechanisms for empowering human autonomy, allowing both patients and health professionals to make informed medical decisions.

Literature in AI supports the role of ‘*transparency and explainability*’ for value creation and engendering user trust.^68–71^ However, telehealth research has provided us with an inadequate view on the role of transparency; specific insights related to emerging technologies are missing. Limited research has explored the role of transparency, with a focus on blockchain technology in telehealth.^72^^,^^73^ Transparency and explainability in AI-enabled health services enhance patient trust and inform clinical decisions.^74–76^ Transparent communication about AI use in health services facilitates patient understanding and trust in diagnoses and treatment recommendations. Understanding how LLMs derive conclusions can enhance trust and acceptance among patients and clinicians. However, a poetical challenge lies in balancing transparency without overwhelming patients. Future research should investigate how to develop user-centered design approaches for LLMs-enabled telehealth use, and explore how to effectively communicate LLMs-generated text for medical recommendation and prediction while preserving patient trust and facilitating informed decision-making. Future research could further enhance our understanding of these crucial responsible-use concepts by employing a critical realist methodology. This approach helps to develop a theory explaining the contextual conditions and mechanisms of transparency that foster users’ trust in LLMs-enabled telehealth services.^77^ From the lens of responsible AI, transparency means being clear about how well LLMs perform and if they have any biases or unfair results due to how they are made and used.

In the landscape of AI ‘fairness’, there is a well-established understanding that diverse technical fairness definitions are mathematically incompatible.^78^ This requires a sociotechnical perspective, including technical components, humanist values, and judgment in each context, to define and contextualize fairness.^79^^,^^80^ The responsible use of telehealth, as discussed in our paper, indeed serves as a compelling experimental ground and context for future research. Researchers can explore the operationalization of technical fairness and provide an understanding of real-world challenges and implications. To promote fairness and minimize bias in telehealth AI systems, future research should investigate methods and bias mitigation mechanisms when LLMs integrated in telehealth systems. Furthermore, exploring how generative AI systems can be made accessible to individuals with disabilities or language and cultural barriers will be essential to ensure this new model of telehealth services are not creating digital divide.^81^

Using LLMs in telehealth services when it generates output based on personal health data could give rise to ‘*privacy*’ concerns. The highly sensitive nature of healthcare data and challenges of data protection in LLMs systems demands responsible use with a focus on data privacy in the telehealth context. Existing literature lacks an in-depth exploration of this challenge, hindering the understanding of effective safeguards for patient information in these emerging landscapes. Academic discourse should address data privacy challenges unique to telehealth and LLMs, including potential health data misuse, improper access, and data breaches. Explicit discussions are crucial to establishing effective privacy protections, safeguarding sensitive health data from misuse, and ensuring responsible use of LMMs in line with the trustworthy AI principles. For instance, potential privacy issues arise in the US context, as physicians use patient data into LLM prompts might inadvertently violate the Health Insurance Portability and Accountability Act (HIPAA), a state-level privacy law.^82^ Even when data does not directly identify individuals, LLMs can still pose risks due to their advanced capabilities.^83^ This shows why it is important to use AI responsibly and protect patient privacy. We see as an opportunity for privacy scholars to explain how proactive privacy protection measures, including specialized training for telehealth providers regarding use of sensitive prompt and data input, along with the restriction of ChatGPT access to trained personnel,^84^ can facilitate minimizing risks of LLMs related data breaches. Responsible use of AI also includes ‘*contestability*’.^85–87^ In our context of research, it relates to stakeholders’ right to challenge and counter decisions made by use of LLMs in telehealth. A promising avenue for future research is to explore the institutional mechanisms of telehealth providers and their responsible responses to telehealth stakeholders (eg, patients and healthcare communities) when they question the use, biases, or improper outcomes of LLMs. For instance, future research should consider how telehealth providers can establish effective mechanisms for contestability (eg, independent review boards). This research avenue will contribute to developing responsible LLMs practices in telehealth use.^20^^,^^88^

### Sociotechnical structural assurance

Structural assurance refers to the presence of protective institutional, and technological structures that guarantee the safety, security, and reliability of conducting business in virtual environments, ensuring a safe and secure experience for users.^89–91^ Building on this, we introduce a contextualized concept of sociotechnical structural assurance. This concept focuses on the assurance of reliability and safety, security, human oversight, and accountability within a socio-technical system, relying on institutional and technological safeguards to create safe and responsible AI-enabled telehealth. In exploring sociotechnical structural assurance, we advocate for diversity of perspectives, particularly from patients and healthcare providers who are the end users of such technology. Moreover, engaging various stakeholders, including digital health officers, security officers, and other healthcare organization roles, will further enhance safety, security, and reliability in LLMs-enabled telehealth.

The ‘*reliability and safety*’ of LLMs and generative AI in telehealth services are crucial for ensuring their responsible use. Reliability and safety failures pose significant risks to the health and well-being of telehealth consumers, potentially compromising the accuracy and appropriateness of generated medical information, interpretation and recommendation provided.^92^^,^^93^ For instance, generated medical texts may prove inaccurate or outdated for telehealth users or patients, impacting the accuracy of their health assessments and patient safety. Furthermore, the recommendations derived from generative AI may exhibit dynamic changes over time. These fluctuations in recommendations stem from factors such as evolving training data, model updates, or stochastic behavior. In the context of LLMs, challenges may arise in accurately making inferences across diverse linguistic contexts, particularly in sensitive domains like medicine where specialized medical knowledge is critical.^94^ Additionally, Generative AI such as ChatGPT often exhibits stochastic behavior, resulting in varying responses to identical queries upon multiple iterations.^95^ This variability could result in potential irreproducible failures, or discrepancies in relation to different population health parameters. These variations can lead to inconsistencies in the use of such information by telehealth providers. Future research should therefore focus on investigating how health professionals involved in the telehealth services can effectively use LLMs in a secure and reliable manner. Additionally, there is a need to investigate information resilience capabilities to address potential errors or inconsistencies that may arise throughout the lifecycle of LLMs in the context of telehealth.

The sensitivity of healthcare data in telehealth, especially when using LLMs, necessitates a focused discussion on ‘*security’* challenges. The integration of LLMs and generative AI in telehealth also introduces security challenges that must be addressed to safeguard sensitive patient data and protect against adversarial attacks. Explicitly addressing these security challenges is essential to sustain responsible use of AI that safeguards healthcare data, engendering institutional trust and helping compliance with data protection regulations and AI Acts. Recent findings from the UK’s National Cyber Security Centre highlighted the emerging risks of prompt injection attacks. These happen when abusers manipulate LLM inputs to create potentially harmful behavior, such as generating offensive content or revealing confidential information.^96^ Additionally, the vulnerability of LLMs to data poisoning attacks poses a significant threat to the security of the models can lead to inaccurate outputs and unreliable decision-making.^97^ These security vulnerabilities demand specialized security measures tailored to the unique context of telehealth. To better understand and mitigate these risks, future research should focus on the development of encryption techniques and access control mechanisms specific to LLMs and generative AI in telehealth settings. Research should also explore methods for early detection and mitigation of prompt injection attacks. Moreover, investigating how telehealth providers can effectively conduct integrity checks to trace the origin of health data inputs and identify potential security breaches will further enhance the security posture of LLMs and generative AI in telehealth service.

Future research in the context of telehealth can explore sociotechnical mechanisms for ensuring adequate ‘*human oversight*’. For example, it would be beneficial to explore methodologies for integrating human oversight into the AI-enabled telehealth process to ensure proper monitoring. Future research can explore the concept of human verification by drawing on perspectives from research on relevance judgment of LLMs.^98^ Doing so through experimental studies, with multiple scenarios of providing telehealth services augmented through LLMs, will prove to be insightful in terms of investigating patient and provider trust and acceptance. Such studies would also identify gaps or tensions between the two stakeholder perspectives. Furthermore, in the context of responsible use of LLMs in telehealth, the concept of use-related activity helps in understanding how individuals interact with LLMs systems within specific telehealth task-technology-patient contexts. Future research should focus on the investigation of use-related activities for ensuring adequate human oversight in LLMs-enabled telehealth. This includes studying the use-related activities of telehealth providers and patients, investigating how they adapt to and interact with LLMs to ensure responsible use. For example, exploring how healthcare professionals communicate and collaborate to understand, monitor, and improve generative AI functionalities, and how patients engage in activities to enhance their knowledge and effective use of generative AI, will contribute to shaping effective human oversight strategies.

In the emerging landscape of LLMs, the identification and allocation of responsibility for the outcomes of these systems is complex.^99^^,^^100^ In the context of telehealth, where the boundaries between telehealth providers, LLMs developers, and regulatory bodies may blur, establishing clear lines of ‘*accountability*’ can be challenging. An avenue for future research is to investigate the tension between ensuring accountability and promoting service innovation in telehealth, as overly restrictive measures may hinder the business value of these systems. The spectrum of AI applications in health service business model including telehealth encompasses various stakeholders, such as software developers, government entities, healthcare units, medical professional organizations, and patient advocacy groups.^101^ Future studies can also explore accountability challenges within this evolving AI landscape and engage all stakeholders in developing an accountability framework, ensuring the responsible use of LLMs in telehealth services. Finally, we excluded the optional ‘expert consultation’ step from the scoping review, considering the evolving nature of the subject. We acknowledge that as the use of LLMs in telehealth matures and practitioners become more familiar, future investigations could incorporate expert consultation for nuanced insights into specific aspects, such as sociotechnical structural assurance, including security, human oversight, and accountability, thereby enriching the scope of the review. Table 4 provides an overview of the discussed research agenda and proposed research questions, focusing on three avenues of investigation, each aligned with the concepts of responsible use illustrated in Table 3.

**Table 4.** An agenda for future research on responsible use of AI.

Research avenues	Concepts	Future research questions
1. Beneficial outcomes	Enhanced health and wellbeing	How can the use of LLMs in telehealth contribute to personalized care, considering factors such as cultural diversity, language barriers, and socio-economic disparities, to enhance the overall telehealth user experience?
		How can healthcare service providers ensure that LLM-enabled telehealth systems adapt to individual patient needs, and how ethical considerations can be implemented to maximize the benefits for both healthcare professionals and patients in the telehealth ecosystem?
		How do LLMs influence the skills and decision-making processes of telehealth providers, and what are the implications for professional development, job satisfaction, and overall performance in the context of AI-enhanced healthcare services?
	Effective use and informed healthcare actions	How do telehealth providers effectively use LLMs to achieve beneficial outcomes for both patients and medical specialists?
		What capabilities can be required to improve the effective use of LLMs for value creation in telehealth service delivery?
	Improved environmental sustainability	In what ways can the integration of LLMs in telehealth services impact the environmental sustainability, and how can AI-enabled services be used to create a balance between efficient healthcare delivery and ecological responsibility.
		How does the responsible deployment of LLMs in telehealth practices impact the overall carbon footprint, considering factors such as reduced patient and clinical staff travel, and the augmentation of medical tasks during virtual consultations?
2. Human-centered values and rights	Human agency	How does the improper automation and over-reliance on LLMs in telehealth impact the autonomy of telehealth providers?
		How can LLMs in telehealth be used to empower health professionals and patients, and what are mechanisms to enhance their autonomy in making informed medical decisions?
		How can healthcare authorities contribute to the establishment of policies and frameworks that safeguarding human agency in telehealth decision-making processes?
	Transparency and explainability	How can healthcare providers implement transparent mechanisms in the use of LLMs-enabled telehealth services to enhance users’ understanding of generative AI processes and potential limitations, fostering public trust in telehealth?
		How do telehealth users perceive and respond to transparent and explainable features in LLMs-enabled telehealth services, and what are the practical implications of these perceptions for enhancing user understanding and control over healthcare decisions?
	Fairness and inclusiveness	How can the design and implementation of telehealth systems incorporating LLMs be optimized to minimize potential biases and foster fairness in healthcare outcomes?
		In what ways can bias mitigation mechanisms be effectively integrated into LLMs-enabled telehealth systems to address unfair outcomes arising from improper design and use?
		How can LLMs-enabled telehealth designers contribute to developing inclusive and accessible approaches within generative AI systems to ensure individuals with disabilities or those facing language and cultural barriers can equally benefit from telehealth services?
	Privacy protection	How do telehealth providers can manage patient data input into LLMs and comply with privacy regulations and acts like the HIPAA and the EU AI act?
		What privacy risks are associated with the use of deidentified data in LLMs within telehealth settings, and how can responsible AI practices mitigate these risks?
		How can proactive measures, such as specialized responsible use training, be implemented to minimize the risks of LLM-related data breaches in telehealth settings?
	Contestability	How can telehealth providers establish process to ensure contestability and address stakeholders’ concerns regarding the inaccurate outcomes and harms of LLMs in telehealth?
		How can telehealth providers navigate the tensions arises in the context of responsible responses from telehealth providers to stakeholders questioning the use of LLMs.
		What are the key challenges faced by telehealth stakeholders, including patients and healthcare communities, when contesting decisions made by LLMs in telehealth, and how can telehealth providers address these challenges responsibly?
3. Sociotechnical structural assurance	Reliability and safety	How can the reliability of LLMs be enhanced in telehealth services to ensure the accuracy and appropriateness of generated medical information for health assessments and patient safety?
		How can health systems designers and telehealth providers address dynamic changes in recommendations derived from LMMs in telehealth, minimizing irreproducible failures and ensuring consistency across different population health parameters?
		How can information resilience capabilities be developed and integrated into telehealth systems to effectively manage and rectify potential errors or inconsistencies that may arise throughout the lifecycle of LLMs, ensuring ongoing reliability and safety?
	Security	How can encryption techniques be tailored to address the unique security challenges posed by the integration of LLMs in telehealth, safeguarding patient data from unauthorized access?
		What methods can be developed to detect and mitigate prompt injection attacks in the context of telehealth, where abusers manipulate LLM inputs to generate potentially harmful behavior or disclose confidential patient information?
		How can healthcare security managers and data protection officers effectively implement integrity checks to trace the origin of health data inputs, enabling early detection of security breaches and ensuring the reliability of outputs from LLMs in telehealth service?
	Human oversight	How effective are human-in-the-loop approaches in ensuring proper oversight in use of LLMs in telehealth, and what are the sociotechnical mechanisms influencing their success?
		To what extent does the concept of human verification, drawing on relevance judgment research of LLMs, contribute to enhancing human oversight in telehealth?
	Accountability	How can clear lines of accountability be established in telehealth, considering the complex interplay between telehealth providers, LLMs developers, and regulatory bodies in the context of AI systems?
		How do varying healthcare stakeholders, including medical AI developers, government entities, healthcare units, and patient advocacy groups, perceive and navigate accountability challenges in use of LLMs in telehealth?
		To what extent do existing organizational accountability practices for responsible use AI in telehealth impact service innovation, and how can a balance be achieved to ensure responsible use of LLMs without hindering telehealth business value?

## Conclusions

This scoping review sheds light on the current landscape of research regarding the application of LLMs in telehealth. The low number of studies emphasizes that more research is needed in this emerging technological domain. By drawing on ethical guidelines for trustworthy AI principles and AI literature, as well as theory of effective use, this paper provides pathways for future research in the context of LLMs within telehealth. These ethical considerations center around health, societal well-being, human-centered values, and sociotechnical structural assurance, providing a foundation for subsequent research. As telehealth continues to advance by using AI, prioritizing the responsible integration of LLMs in telehealth becomes essential, ensuring ethical practices, safety, patient privacy, and the realization of beneficial healthcare outcomes.

## Data Availability

This article is a review and perspective piece; all referenced articles included in the review are cited in the table in the results section, and there is no associated empirical data.
